# Dexmedetomidine is neuroprotective in an *in vitro *model for traumatic brain injury

**DOI:** 10.1186/1471-2377-12-20

**Published:** 2012-04-11

**Authors:** Marc Schoeler, Philip D Loetscher, Rolf Rossaint, Astrid V Fahlenkamp, Georg Eberhardt, Steffen Rex, Joachim Weis, Mark Coburn

**Affiliations:** 1 Department of Anesthesiology, University Hospital of the RWTH Aachen, Pauwelsstraße 30, 52074 Aachen, Germany; 2 Institute of Neuropathology, University Hospital of the RWTH Aachen, Pauwelsstraße 30, 52074 Aachen, Germany; 3 Department of Anesthesiology, University Hospital of the KU Leuven, Herestraat 49, 3000 Leuven, Belgium

**Keywords:** Neuroprotection, Traumatic brain injury, Dexmedetomidine

## Abstract

**Background:**

The α_2_-adrenoreceptor agonist dexmedetomidine is known to provide neuroprotection under ischemic conditions. In this study we investigated whether dexmedetomidine has a protective effect in an *in vitro *model for traumatic brain injury.

**Methods:**

Organotypic hippocampal slice cultures were subjected to a focal mechanical trauma and then exposed to varying concentrations of dexmedetomidine. After 72 h cell injury was assessed using propidium iodide. In addition, the effects of delayed dexmedetomidine application, of hypothermia and canonical signalling pathway inhibitors were examined.

**Results:**

Dexmedetomidine showed a protective effect on traumatically injured hippocampal cells with a maximum effect at a dosage of 1 μM. This effect was partially reversed by the simultaneous administration of the ERK inhibitor PD98059.

**Conclusion:**

In this TBI model dexmedetomidine had a significant neuroprotective effect. Our results indicate that activation of ERK might be involved in mediating this effect.

## Background

In the United States an estimated 1.1 million patients are treated for traumatic brain injury (TBI) annually [1]. Of these, 124.000 patients suffer from long-term disabilities and 50.000 die, which makes TBI a major cause of premature death [1,2]. In addition, long term medical care and loss of income create a considerable social and economical burden, which remains an unsolved public health problem [3]. Regrettably, to date there exists no clinically established therapy targeting the mechanisms involved in TBI and treatment remains limited to symptomatic approaches.

Dexmedetomidine is a highly selective α_2_-adrenoreceptor agonist with sedative, analgesic and sympatholytic properties. It was developed and investigated during the 1980s and 1990s and was initially used to achieve sedation and analgesia in veterinary medicine. In 1999 it was approved for use in humans by the US Food and Drug Administration for short-term sedation (< 24 h) of patients in intensive care units and non-intubated patients prior to and during surgical procedures. Recently, dexmedetomidine was approved in Europe for sedation of adult intensive care unit patients requiring a sedation level not deeper than arousal in response to verbal stimulation.

Besides dexmedetomidine's well-proven sedative effects an increasing body of both *in vitro *and *in vivo *evidence indicates that dexmedetomidine also exerts a cell-protective effect on nervous tissue under ischemic conditions [4-7]. There is recent evidence suggesting that this effect is mediated not only by dexmedetomidine's α_2_-agonistic properties, but also by the binding to imidazoline I_1_-recepors [8]. The signal transduction cascade linked to these receptors comprises extracellular signal-regulated protein kinase 1 and 2 (ERK 1&2) and is known to be an important regulator for cell survival and mediator of neuroprotective effects of various agents [9-11]. Nevertheless it has yet to be determined whether dexmedetomidine also promotes survival of nervous tissue that has suffered traumatic injury.

In this study we tested the hypothesis that dexmedetomidine has a protective effect on traumatically injured brain tissue as well. We used organotypic hippocampal brain-slice cultures subjected to a focal mechanical trauma as an *in vitro *model of TBI to analyse a potential neuroprotective effect of dexmedetomidine in TBI.

## Methods

All experiments in the course of this study were approved by the animal protection representative at the Institute of Animal Research at RWTH Aachen University Hospital, according to the German animal protection law §4, Section 3. Unless otherwise stated all chemicals were obtained from PAA Laboratories GmbH (Pasching, Austria).

### Hippocampal slice culture

The organotypic hippocampal slice cultures were prepared from brains of 5-7 day old C57/BL6 mice pups using a well-established technique [12-14]. After decapitation of the pups the brains were quickly extracted from the sculls and placed into ice cold preparation medium comprising Gey's balanced salt solution (Sigma Aldrich, Steinheim, Germany), 5 mg/ml D-(+)-glucose (Roth, Karlsruhe, Germany) and 0.1% antibiotic/antimycotic solution (containing penicillin G 10,000 U/ml, streptomycin sulphate 10 mg/ml and amphotericin B 25 μg/ml).

The hippocampi were then dissected from the brains under stereomicroscopic vision, cut into 400 μm thick slices and dispersed onto the semi-permeable membrane of MilliCell tissue culture inserts (MilliCell-CM, Millipore Corporation, Billerica, MA, USA). These inserts were placed inside 35 mm tissue culture plates (Sarstedt, Newton, MA, USA) and 1 ml of growth medium (50% Eagle minimal essential medium with Earle's salts, 25% Hank's balanced salt solution (Sigma-Aldrich, Steinheim, Germany), 25% heat inactivated horse serum, 2 mM L-glutamine, 5 mg/ml D-glucose, 1% antibiotic/antimycotic solution and 50 mM HEPES (hydroxyethyl-piperazine-ethanesulfonic acid) buffer solution (Sigma-Aldrich, Munich, Germany), titrated to pH 7.2) was instilled under the membranes to ensure optimal nutrition of the slices. The slices were then incubated for 14 days at 37°C and a moist atmosphere of 95% air and 5% CO_2_. The growth medium was replaced 24 h after preparation and on every third day thereafter.

### Traumatic brain injury protocol

On the day of the experiment growth medium was exchanged for experimental medium containing 4.5 μM propidium Iodide. Experimental medium differed from growth medium only in the substitution of horse serum for more Eagle minimal essential medium assuring a standardized experimental environment for all slices. The slices were then incubated for 30 min at 37°C with a moist atmosphere of 95% air and 5% CO_2 _before capturing baseline fluorescence images. Slices were then traumatized using a specially designed apparatus that ensured an equal and reproducible trauma during the course of the experiments [15,16]. For this purpose a metal stylus was lifted 7 mm above the slice with the aid of an electromagnet. When power to the magnet was switched off the stylus fell onto the slices producing a mechanical trauma of 5.26 μJ onto the hippocampus's CA1 region. The stylus was retrieved immediately after the impact and experimental medium was exchanged for new experimental medium in those plates belonging to the positive control group. In the experimental groups dexmedetomidine (Precedex^® ^100 μg/ml Dexmedetomidine, Orion Pharma, Espoo, Finland) was added to this experimental medium in various concentrations (100 μM, 10 μM, 5 μM, 1 μM, 0.1 μM, 0.01 μM). Concentrations were prepared based upon a molar weight of 236.7 g/mol for dexmedetomidine-HCl as a basis. For the negative control group (non-traumatized slices), data from a previous, methodically identical study was used [12].

To further investigate the protective properties of dexmedetomidine, a series of other experiments was performed using a 1 μM concentration which in the dose-finding study had been proven to be the most effective dose. To assess possible neuroprotective effects in a delayed application, experiments were repeated for 1 μM dexmedetomidine, exchanging experimental medium at 2 and 3 h *after *the trauma, respectively. In order to verify the hypothesis that dexmedetomidine's neuroprotective properties involve activation of ERK the mitogen-activated/extracellular signal-regulated protein kinase kinase 1 (MEK1) -inhibitor PD98059 (Cell Signaling Technology, Danvers, MA, USA) was added in a 5 μM concentration to the experimental medium containing 1 μM dexmedetomidine.

All slices were incubated for 72 h at 37°C and a moist atmosphere of 95% air and 5% CO_2 _after trauma.

In order to compare the effects of dexmedetomidine on cell survival with those of hypothermia, a positive control group of slices was incubated at 32° and a moist atmosphere of 95% air and 5% CO_2 _for 72 h after trauma. An experimental group exposed to 1 μM of Dexmedetomidine was also incubated at 32°C for 72 h after trauma to study a possible synergistic effect of dexmedetomidine and hypothermia.

### Quantification of cell death and statistical analysis

After the incubation period final fluorescence images were taken.

To quantify cell death propidium iodide was present in the media throughout all experiments. Propidium iodide is a widely used dyeing agent able to bind to nuclear DNA in a non-specific manner. Upon binding it becomes highly fluorescent with a peak emission in the spectrum of visible red light. Its properties render it incapable of penetrating intact phospholipids bilayers such as the cell membrane and the nuclear membrane. However, if the integrity of the cellular membranes is disrupted as in apoptotic or necrotic processes, propidium iodide can diffuse into the nucleus and stain the DNA.

Baseline and final fluorescence images were taken using an upright fluorescence microscope (Zeiss Axioplan, Carl Zeiss MicroImaging GmbH, Jena, Germany) equipped with a rhodamine filter and a low-power x4 objective lens (Zeiss Achroplan 4x/0.10, Carl Zeiss MicroImaging GmbH, Jena, Germany) and captured with a digital camera (SPOT Pursuit 4 MP Slider, Diagnostic Instruments Inc, Sterling Heights, MI, USA) and applicable software.

To eliminate any influence of the mercury lamp's fluctuating intensity, the exposure time was adapted before each imaging session using a standard fluorescence slice (Fluor-Ref, Omega Optical, Brattleboro, VT, USA) as reference.

**Figure 1:**
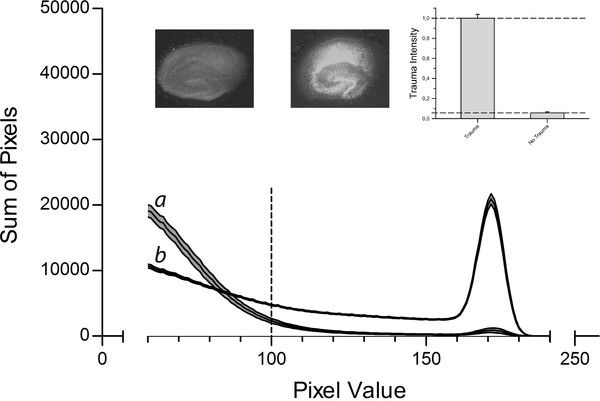
**Control Data**. Slices were cultivated for 14 days before being subjected to TBI introduced by a stylus dropped onto the CA1 region. Slices were then incubated in an atmosphere of 95% air and 5% CO_2_. Negative control group slices were treated the same way, except for the trauma. After 72 h trauma intensity was assessed by fluorescence imaging and pixel based analysis. The fluorescence intensity corresponded to the trauma intensity of each pixel and was broken down to a value between 0 and 255. The histogram shows the distribution of pixels for the different fluorescence intensities for the negative control group (n = 35, labelled *a*) and the slices of the positive control group (n = 185, labelled *b*). The middle line represents the mean for each fluorescence value; the upper and lower lines represent the upper and lower boundaries of the SEM. The interrupted vertical line is the applied threshold at a grey scale value of 100. The sum of all pixels beyond this threshold was calculated for each group and served as a measure for trauma intensity. Above the graph, example images for the negative control group (left) and positive control group (right) are shown. The insert in the upper right corner represents the trauma levels of both control groups normalized to the positive control group.

ImageJ software was used to analyze the images. The fluorescence intensity of each pixel of the red channel was broken down to an eight bit value (between 0 and 255). Areas with high fluorescence values were equivalent to high propidium iodide uptake, thus strong cell damage. A histogram was then created showing the distribution of intensities within the image. Results of previously conducted studies using the same method [12,13,17] point out that values below a threshold of 100 represent background fluorescence. Therefore, only values beyond this threshold were summarized and this integral served as a quantitative measure for cell death (Figure 1).

### Statistical analysis

Mean ± SEM (standard error of the mean) was calculated for each group using SPSS 19 (IBM^® ^SPSS^® ^Statistics, IBM Corporation, Somers, NY, USA). The fluorescence value of the positive control group was set to unity (1 ± 0.037) and served as a reference to put the observed effect of dexmedetomidine on cell survival into perspective. Values of all other groups were normalized to the positive-control group. Statistical relevance of the results was assessed performing an analysis of variance (ANOVA). *P *≤ 0.05 was considered statistically significant.

## Results

**Figure 2:**
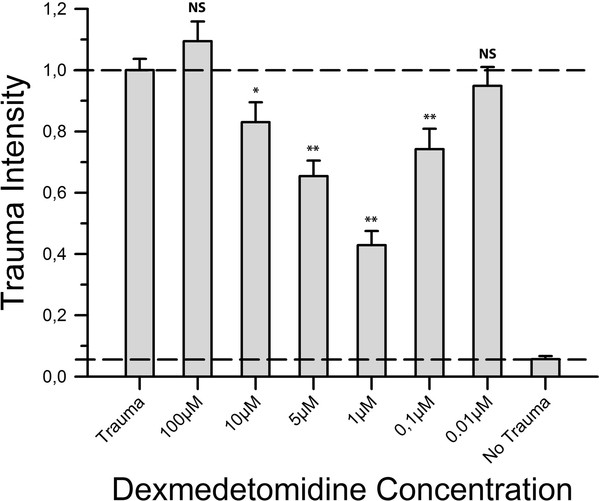
**Neuroprotective effect of dexmedetomidine**. After trauma, slices were exposed to varying concentrations of dexmedetomidine. Following 72 h of incubation fluorescence images were taken and analysed. For each group an average of 52 slices with a minimum of 39 slices was used. Trauma intensities of the different groups are shown in relation to trauma intensity in the positive control group (n = 185). Trauma intensity was significantly lower in the groups 10 μM, 5 μM, 1 μM and 0.1 μM (*p ≤ 0.05, **p ≤ 0.001) when compared to the positive control group. In contrast, the groups 100 μM and 0.01 μM showed no statistical difference compared to the positive control group (*p *= 0.213 and *p *= 0.485, respectively).

As Figure 2 points out the concentrations 10 μM, 5 μM, 1 μM and 0.1 μM showed a significant reduction in trauma intensity with a maximum effect at 1 μM, where the fluorescent value was 42.9% of the positive control group's value. No significant change in trauma intensity was observed at 100 μM (1.094 ± 0.065 vs. 1 ± 0.037, *P *= 0.213) and 0.01 μM (0.949 ± 0.061 vs. 1 ± 0.037, *P *= 0.485).

**Figure 3:**
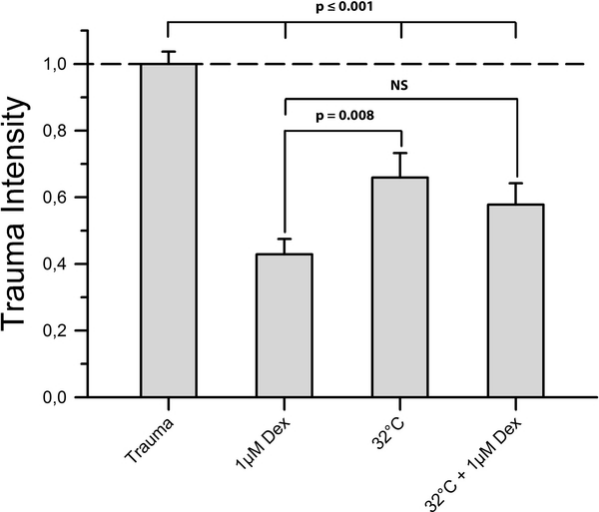
**Hypothermia**. We subjected a group of slices (n = 49) to mild hypothermia of 32°C for 72 h after TBI to distinguish the observed dexmedetomidine protection from already determined neuroprotective treatment. Furthermore, we treated another group of slices (n = 68) simultaneously with both mild hypothermia and 1 μM dexmedetomidine to investigate a potential additive effect of both treatments. Figure 3 shows that treatment with mild hypothermia caused a significant reduction of trauma intensity when compared to the positive control group (*p *≤ 0.001). However, this protective effect was significantly lower than protection provided by 1 μM dexmedetomidine (*p *= 0.008). Treatment with both hypothermia and 1 μM dexmedetomidine proved to be more effective in reducing trauma than hypothermia alone (*p *= 0.415) but showed no statistical difference compared to a treatment with 1 μM dexmedetomidine alone (*p *= 0.073).

While hypothermia proved to be neuroprotective when comparing its effects to the positive control group (0.659 ± 0.074 vs. 1 ± 0.037, *P *< 0.001), hypothermia was associated with significantly less neuroprotection than dexmedetomidine at a concentration of 1 μM (0.659 ± 0.074 vs. 0.429 ± 0.046, *P *= 0.008). The simultaneous application of hypothermia and 1 μM dexmedetomidine was neither significantly superior nor inferior to the application of 1 μM dexmedetomidine alone (0.578 ± 0.064 vs. 0.429 ± 0.046, *P *= 0.073) (Figure 3).

**Figure 4:**
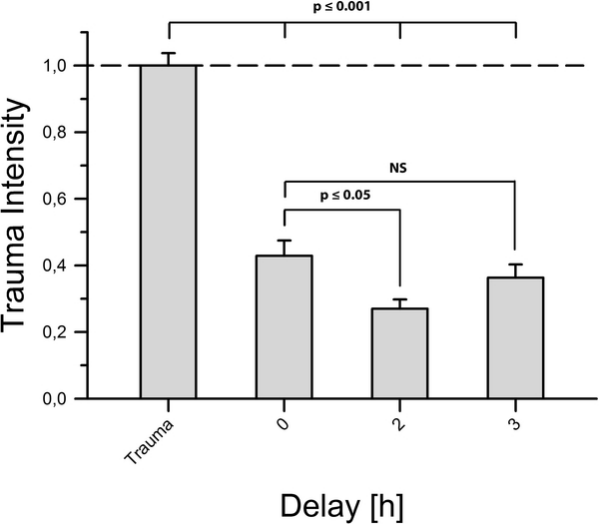
**Delayed application of dexmedetomidine**. Here, we applied dexmedetomidine 2 and 3 h after TBI in a concentration of 1 μM. The two-hour delay group contained n = 24 slices and the three-hour delay group contained n = 31 slices. We found that both groups showed a level of trauma intensity significantly lower than the positive control group's (*p *≤ 0.001). An application delayed by 2 h appeared to protect slices even better than immediate application of dexmedetomidine (*p *≤ 0.05). After a delay of 3 h no statistical difference from an immediate application was observed (*p *= 0.335).

Also, the delayed application of dexmedetomidine (2 h and 3 h after trauma, respectively) showed a protective effect. Application of dexmedetomidine 2 h after trauma proved even more efficient than immediate application (0.27 ± 0.028 vs. 0.429 ± 0.046, *P *= 0.030) while a delay of three hours (0.363 ± 0.04 vs. 0.429 ± 0.046, *P *= 0.335) resulted in similar effects as the instantaneous application (Figure 4).

**Figure 5:**
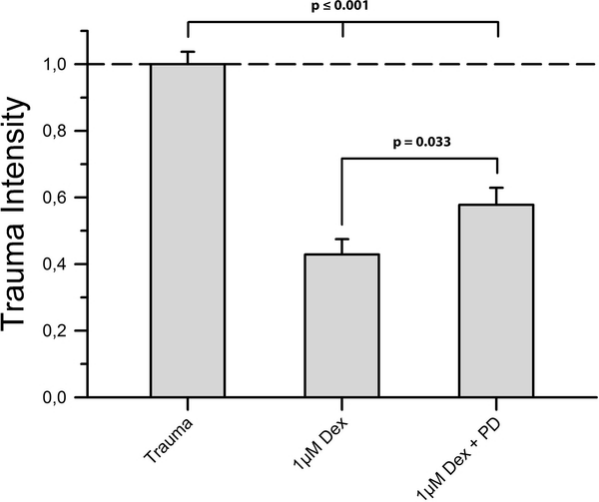
**MEK-Inhibitor**. To investigate whether the ERK1/2-pathway is involved in the mediation of dexmedetomidine's neuroprotective properties towards mechanically injured brain tissue we applied PD98059, inhibitor of ERK1/2's direct activator MEK1, in a 5 μM concentration along with 1 μM dexmedetomidine immediately after trauma. Here, n = 46 slices were subjected to the aforementioned conditions. As shown in Figure 5 trauma intensity was significantly higher in the inhibitor group than in the dexmedetomidine group (*p *= 0.033) but still appreciably lower than in the positive control group (*p *≤ 0.001).

The simultaneous application of 1 μM dexmedetomidine and PD98059 resulted in a statistically significant reduction of cellular protection (0.578 ± 0.051 vs. 0.429 ± 0.046, *P *= 0.033) (Figure 5).

## Discussion

The aim of this study was to investigate the effects of the α_2_-adrenoreceptor agonist dexmedetomidine on cell survival in an established *in vitro *model for TBI. Using organotypic hippocampal slice cultures subjected to a focal mechanical trauma we found that dexmedetomidine had a dose-dependent protective effect on hippocampal cells. The dose-effect curve was U-shaped, with a concentration of 1 μM showing the strongest effect. Moreover, we observed neuroprotection by dexmedetomidine even when applied with a delay after the onset of the traumatic injury. Apparently, the neuroprotective effects of dexmedetomidine are mediated - at least in part - by ERK as they were abolished by the co-administration of PD98059. Interestingly, the co-application of hypothermia and dexmedetomidine did not exert synergistic effects.

We treated experimental groups with varying concentrations of dexmedetomidine after trauma. Results show that a concentration of 1 μM dexmedetomidine was most effective at reducing trauma intensity. This finding is consistent with data from a recent study using hippocampal slice culture subjected to oxygen and glucose deprivation [5]. Further dilution as well as a higher concentration of dexmedetomidine provided lower protection until finally, at 100 μM and 0.01 μM, no difference compared to the positive control group was observed. Comparable observations have been made in other studies investigating dexmedetomidine's protective properties [5,18].

Dexmedetomidine's neuroprotective properties have largely been attributed to its agonist actions at α_2_-adrenoreceptors [19,20]. Although recent investigations point out that its interaction with imidazoline I1 receptors and the activation of ERK might also play a role [5,8,21] we did not explicitly analyze imidazoline I_1 _receptors. However, the results of our experiments indicate that activation of ERK is an important factor in the protection of traumatized nervous tissue by dexmedetomidine. ERK is a key enzyme in cell metabolism activated by many different types of tissue injury and has been attributed a "survival"- function. The inhibition of a survival signals itself can cause cells to undergo apoptosis; therefore application of PD98059, inhibitor of ERK's direct activator MEK1, might as well have had a negative effect on cellular survival that superposed with the positive effect of dexmedetomidine without specifically counteracting dexmedetomidine. To rule out this possibility western blots could have been performed. Comparison of levels of activated ERK for the positive control group, the 1 μM dexmedetomidine group and the 1 μM dexmedetomidine + PD98059 group could have backed up our hypothesis. This has recently been performed in a similarly designed study investigating dexmedetomidine's neuroprotective properties using organotypic hippocampal slice cultures subjected to oxygen and glucose deprivation. Results of this study showed that dexmedetomidine's and PD98059's effects do not simply superpose but that both act as two opponents on the same cascade [5]. We therefore decided not to perform these tests and interpreted our finding as a sufficient hint towards an involvement of ERK in the mediation of the brain protective properties of dexmedetomidine in TBI.

Many experimental studies have revealed neuroprotective properties of hypothermia [13,16,22,23] and the advantages of hypothermia in certain clinical circumstances have been demonstrated [24]. To put our findings into context we subjected a group of slices to mild hypothermia of 32°C from trauma until final imaging. As expected, hypothermia alone did prove to be strongly protective, but 1 μM dexmedetomidine was even more effective at reducing trauma intensity. Interestingly, as described before in *in vivo *models of incomplete cerebral ischemia, a combination of hypothermia and 1 μM dexmedetomidine did not result in synergistic effects [7].

In a clinical setting, unpredictability is in the nature of TBI and application of any specific therapy will inevitably be delayed. Therefore we investigated the effect of dexmedetomidine when applied 2 or 3 h after trauma. In our model dexmedetomidine proved to be even more efficient when application was delayed by 2 h. However, by delaying application for 3 h no appreciable difference could be observed any more compared to immediate application.

We acknowledge that our results should be interpreted within the context of several limitations.

Propidium iodide labels the DNA of any disrupted cell and we were therefore not able to distinguish between necrosis and apoptosis. However, we aimed to quantify the total amount of cell death and it is agreed that propidium iodide uptake correlates well with the number of damaged cells [15,25].

We opted for organotypic slice cultures because this model allows easy access to *in vitro *manipulation of nervous tissue and yet mimics closely the *in vivo *state of the tissue with respect to morphological and functional characteristics [26,27]. Concerning the extrapolation to an *in vivo *situation, results from organotypic slice cultures have been demonstrated to be a satisfying compromise between dissociated cell culture and *in vivo *models using whole animals [28,29]. We admit, however, that extrapolation of our results to the *in vivo *situation is complex. In a living organism, the development and the outcome from TBI is significantly affected by a plethora of different variables that can only partly, if at all, be taken into consideration in an *in vitro *model (e.g. cerebral perfusion pressure, edema of surrounding tissue, focal or global cerebral ischemia). Such conditions may be affected by dexmedetomidine independently from its direct effects on neuronal survival. Despite these disadvantages we believe that our model is appropriate as it focuses on the mechanical component of the injury and allows the analysis of the intrinsic neuroprotective properties of a drug independently from its possible interactions with confounding variables that can only poorly be controlled in an *in vivo *setting.

## Conclusion

We have found that dexmedetomidine has a protective effect on hippocampal slice cultures subjected to a focal mechanical trauma. The maximum effect was observed at 1 μM, with the observed trauma reduction being significantly more pronounced than observed in slices treated with hypothermia. Trauma intensity did not further decline when applying dexmedetomidine and hypothermia simultaneously. Also, dexmedetomidine's effect was partially reversed by simultaneous application of MEK1 Inhibitor PD98059 indicating the involvement of the ERK-signalling cascade.

## Key messages

• Dexmedetomidine exerts a neuroprotective effect when administered after TBI in this model of organotypic hippocampal slice cultures

• The protective effect of dexmedetomidine is significantly stronger than the effect of mild hypothermia at 32°C

• Dexmedetomidine has a protective effect even if application after TBI is delayed by 2 and 3 h, respectively

• Activation of ERK might be involved in mediating Dexmedetomidine's protective effect

## Abbreviations

TBI: Traumatic brain injury; ERK: Extracellular signal-regulated protein kinase; HEPES: Hydroxyethyl-piperazine-ethanesulfonic acid; MEK: Mitogen-activated/extracellular signal-regulated protein kinase kinase; SEM: Standard error of the mean; ANOVA: Analysis of variance.

## Competing interests

This study was supported by Orion Corporation, Espoo, Finland.

RR received consultant fees from Orion Corporation, a company producing and marketing pharmaceuticals such as dexmedetomidine. All other authors declare that they have no competing interests.

## Authors' contributions

MS conducted the experimental laboratory work, performed the statistical analysis and drafted the manuscript. AVF, GE, RR and SR participated in the study design and coordination and helped to draft the manuscript. PDL and JW helped to perform the study and assisted in drafting the manuscript. MC originated the study, participated in its study design and coordination and helped to draft the manuscript. All authors read and approved the final text.

## Pre-publication history

The pre-publication history for this paper can be accessed here:

http://www.biomedcentral.com/1471-2377/12/20/prepub
